# Risk Factors for Non-Adherence to cART in Immigrants with HIV Living in the Netherlands: Results from the ROtterdam ADherence (ROAD) Project

**DOI:** 10.1371/journal.pone.0162800

**Published:** 2016-10-05

**Authors:** Sabrina K. Been, David A. M. C. van de Vijver, Pythia T. Nieuwkerk, Inês Brito, Sarah E. Stutterheim, Arjan E. R. Bos, Mireille E. G. Wolfers, Katalin Pogány, Annelies Verbon

**Affiliations:** 1 Department of Internal Medicine, Erasmus University Medical Centre, Rotterdam, The Netherlands; 2 Department of Virology, Erasmus University Medical Centre, Rotterdam, The Netherlands; 3 Department of Medical Psychology, Academic Medical Centre, Amsterdam, The Netherlands; 4 Faculty of Psychology and Educational Sciences, Open University of the Netherlands, Heerlen, the Netherlands; 5 Municipal Public Health Service Rotterdam-Rijnmond, Infectious Disease Control Division, Rotterdam, The Netherlands; 6 Department of Internal Medicine, Maasstad Hospital, Rotterdam, The Netherlands; Azienda Ospedaliera Universitaria di Perugia, ITALY

## Abstract

In the Netherlands, immigrant people living with HIV (PLWH) have poorer psychological and treatment outcomes than Dutch PLWH. This cross-sectional field study examined risk factors for non-adherence to combination Antiretroviral Therapy (cART) among immigrant PLWH. First and second generation immigrant PLWH attending outpatient clinics at two HIV-treatment centers in Rotterdam were selected for this study. Socio-demographic and clinical characteristics for all eligible participants were collected from an existing database. Trained interviewers subsequently completed questionnaires together with consenting participants (*n* = 352) to gather additional data on socio-demographic characteristics, psychosocial variables, and self-reported adherence to cART. Univariable and multivariable logistic regression analyses were conducted among 301 participants who had used cART ≥6 months prior to inclusion. Independent risk factors for self-reported non-adherence were (I) not having attended formal education or only primary school (OR = 3.25; 95% CI: 1.28–8.26, versus University), (II) experiencing low levels of social support (OR = 2.56; 95% CI: 1.37–4.82), and (III) reporting low treatment adherence self-efficacy (OR = 2.99; 95% CI: 1.59–5.64). Additionally, HIV-RNA >50 copies/ml and internalized HIV-related stigma were marginally associated (*P*<0.10) with non-adherence (OR = 2.53; 95% CI: 0.91–7.06 and OR = 1.82; 95% CI: 0.97–3.43). The findings that low educational attainment, lack of social support, and low treatment adherence self-efficacy are associated with non-adherence point to the need for tailored supportive interventions. Establishing contact with peer immigrant PLWH who serve as role models might be a successful intervention for this specific population.

## Introduction

In the Netherlands, more than 40% of the 17,750 HIV patients enrolled in clinical care are immigrants, with 86% of these immigrant people living with HIV (PLWH) originating from outside of Western Europe [1]. Immigrant PLWH can therefore be considered a ‘key population’ in Dutch HIV care. Despite an awareness that high levels of adherence to combination Antiretroviral Therapy (cART) are crucial for virological suppression and subsequent beneficial clinical outcomes [2, 3], previous research has shown that immigrant PLWH originating from countries outside of Western Europe have poorer treatment and health outcomes than Dutch PLWH. In fact, when treated with cART, immigrant PLWH less frequently reach virological suppression and more frequently experience virological failure [4–7]. This may be attributable to a later diagnosis [8] and/or poor treatment adherence [6, 9–11]. Treatment adherence has been found to be associated with a number of psychosocial factors including depressive symptoms, internalized HIV-related stigma, disclosure concerns, quality of life, and social support [12–14] and previous research has shown that immigrant PLWH tend to experience more depression, more internalized stigma, more disclosure concerns, less quality of life, and less social support than Dutch PLWH [6, 15].

In this study, we assessed socio-demographic and psychosocial risk factors for non-adherence to cART in immigrant PLWH enrolled in clinical care. Understanding risk factors for non-adherence is important as this knowledge can serve as input for interventions aiming to improve adherence, and subsequently clinical and psychosocial outcomes, among immigrant PLWH in the Netherlands.

## Methods

### Participants and procedure

First and second generation immigrants with HIV, aged 18 or older, were eligible for inclusion in this study. Participants born outside of Western Europe were categorized as first generation immigrants and participants for whom one or both parents were born outside of Western Europe were categorized as second generation immigrants. In order to participate, participants had to be sufficiently fluent in at least one of the following languages: Dutch, English, French, Spanish, or Portuguese.

Following medical ethics approval from the Medical Ethics Review Committee of the Erasmus University Medical Centre, eligible participants were recruited at outpatient clinics in two Dutch HIV treatment centers (i.e., Erasmus University Medical Centre and Maasstad Hospital) between November of 2012 and July of 2013. Eligible participants were selected consecutively during their regular visits by their treating physician or HIV nurse. Information regarding gender, date of birth, country of birth, most recent HIV-RNA value and CD4 cell count (with a maximum of 46 days after their planned visit), and combination Antiretroviral Therapy (cART) start date were collected from the ATHENA observational HIV cohort database (the Dutch national HIV registry of HIV treatment centers). When incomplete, data were cross checked with medical records. Eligible participants were defined as ‘cART experienced’ if they had started treatment more than six months prior to inclusion. A plasma HIV-1 or HIV-2 RNA of >50 copies/ml was defined as ‘detectable’. Trained interviewers completed a questionnaire together with the participants, who all provided written informed consent prior to participation.

### Questionnaire

Via the questionnaire, data measuring additional relevant socio-demographic characteristics, psychosocial variables, and self-reported adherence to cART were collected. Items regarding alcohol and drug use in the past 30 days prior to questionnaire completion were retrieved from the EuropASI [16].

Region of origin was determined by first determining country of birth for first generation immigrants and the parent(s)’(s) country of birth for second generation immigrants and then categorizing those countries into the following regions: Sub Saharan Africa, the Caribbean, Latin America, or other. The ‘other’ category comprises Central, Eastern, and Southern Europe, North Africa, Middle East, South Asia, East Asia, the Pacific region, Australia, New Zealand, and North America. If both parents were immigrants and originated from different regions, the participants`region of origin was based on the mother`s region of origin.

Social support was assessed using the eight-item modified Medical Outcomes Study Social Support Survey (mMOS-SS)[17]. This scale is a widely used, and considered a reliable and valid measure of social support. It contains two subscales, one that assesses instrumental social support (e.g., ‘If you needed it, how often is someone available to prepare your meals if you are unable to do it yourself?’) and one assessing emotional social support (e.g., ‘…to turn to for suggestions about how to deal with a personal problem?’). Answers were provided on a 5-point Likert scale ranging from 1 = ‘never’ to 5 = ‘always’. Internal reliability of the mMOS-SS in this study was measured by Cronbach’s alpha (α = 0.89, Table 1). The average of the scores of both subscales was subsequently transformed to a 0–100 scale [18] with higher scores indicating greater social support.

**Table 1:** Internal consistency analyses.

Variable domain	Number of items	Scoring range	N	Cronbach’s α
**mMOS-SS**	8	8–40	332	0.89
*Instrumental social support*	4		341	0.91
*Emotional social support*	4		339	0.77
**IA-RSS**	6	6–36	333	0.79
**HIV-ASES**	12	0–120	286	0.87
*Integration*	9		302	0.83
*Perseverance*	3		300	0.58
**SF-12**	12		346	0.83
*Physical QoL*	6	0–100	348	0.77
*Mental QoL*	6	0–100	346	0.76
**Adherence**	4		293	0.69

mMOS-SS, modified Medical Outcomes Study Social Support Survey; IA-RSS, Internalized AIDS-Related Stigma Scale; HIV ASES, HIV Treatment Adherence Self-Efficacy Scale; SF-12, 12-item Short Form Health Survey.

Internalized HIV-related stigma was assessed using the six-item Internalized AIDS-Related Stigma Scale (IA-RSS) which has previously been validated in samples of PLWH in Sub Saharan Africa [19]. An example item is: ‘Being HIV positive makes me feel dirty’. Because previous research has shown that participants often struggle with binary answer options, we diverged from the original binary answer options (agree/disagree) and expanded the number of answer options to four ranging from 1 = ‘strongly disagree’ to 4 = ‘strongly agree’. Higher scores were considered indicative of more internalized HIV-related stigma. The IA-RSS demonstrated sufficient reliability in this study (α = 0.79, Table 1).

Self-efficacy for HIV treatment adherence was assessed using the HIV Treatment Adherence Self-Efficacy Scale (HIV-ASES). This validated and widely used 12-item scale contains items measuring the integration of HIV treatment (e.g., ‘In the past month, how confident have you been that you can integrate your treatment into your daily routine?’) and perseverance (e.g., ‘…continue with your treatment even when you are feeling discouraged about your health?’). Answers were provided on a 10-point scale with higher sores indicating greater perceived self-efficacy [20]. In this study, Cronbach’s α was 0.87 (Table 1).

Physical and mental quality of life was measured using the 12-item Short Form Health Survey (SF-12) [21]. This 12-item scale is validated across age, medical condition, and treatment groups and results are expressed as two meta-scores: the Physical Component Summary (PCS) and the Mental Component Summary (MCS). Higher scores indicate a higher quality of life. Cronbach`s α in our study was 0.77 for the PCS and 0.76 for the MCS (Table 1).

Self-reported adherence to cART was assessed by four items developed in previous studies of adherence (α = 0.69, Table 1) (S1 Table). Two of the items measured adherence beliefs: Q1: ‘Thinking about the past four weeks, how would you rate your ability to take all your medications as your doctor prescribed them?’ (answers scored on a 6-point Likert scale ranging from 1 = ‘very poor’ to 6 = ‘excellent’) and Q2: ‘Thinking about the past four weeks, how often did you take all your HIV antiretroviral medications as your doctor prescribed them?’ (answers scored on a 5-point Likert scale ranging from 1 = ‘none of the time’ to 5 = ‘all of the time’)[22]. One item measured complete adherence in the previous week: Q3: ‘How many days in the past week did you take all anti-HIV medicines that were prescribed?’ (answers scored on a 5-point Likert scale ranging from 1 = ‘not one day’ to 5 = ‘all 7 days’)[23–25]. The final item measured the most recent missed dose: Q4: ‘When was the last time you missed any of your anti-HIV medications?’ (answers scored on a 6-point Likert scale ranging from 1 = ‘within the past week’ to 6 = ‘never missed’)[26]. Participants were classified as ‘non-adherent’ if they responded with ‘very poor’ to ‘good’ to Q1, ‘none of the time’ to ‘most of the time’ to Q2, ‘not one day’ to ‘5 or 6 days’ to Q3, and ‘within the past week’ to ‘2–4 weeks ago’ to Q4 (S2 Table). We also conducted analyses using both stricter and less strict criteria for adherence (S2 Table) and these results are presented in S3 and S4 Tables.

### Statistical analysis

Chi^2^ and Fisher-Freeman-Halton Exact tests were used to compare categorical data between groups while T-tests and the Mann-Whitney tests where used to compare continuous data, when appropriate. Logistic regression analyses were then conducted to determine independent risk factors for self-reported non-adherence in cART experienced participants. For these analyses, scores from the psychosocial variables (social support, internalized HIV-related stigma, treatment adherence self-efficacy, and physical and mental quality of life) were dichotomized based on their median values: <75, >15, <105, <52, and <48.5. In multivariable analysis, we included all variables demonstrating a *P*<0.15 in the univariable analyses.

## Results

### Participant characteristics

**Fig 1:**
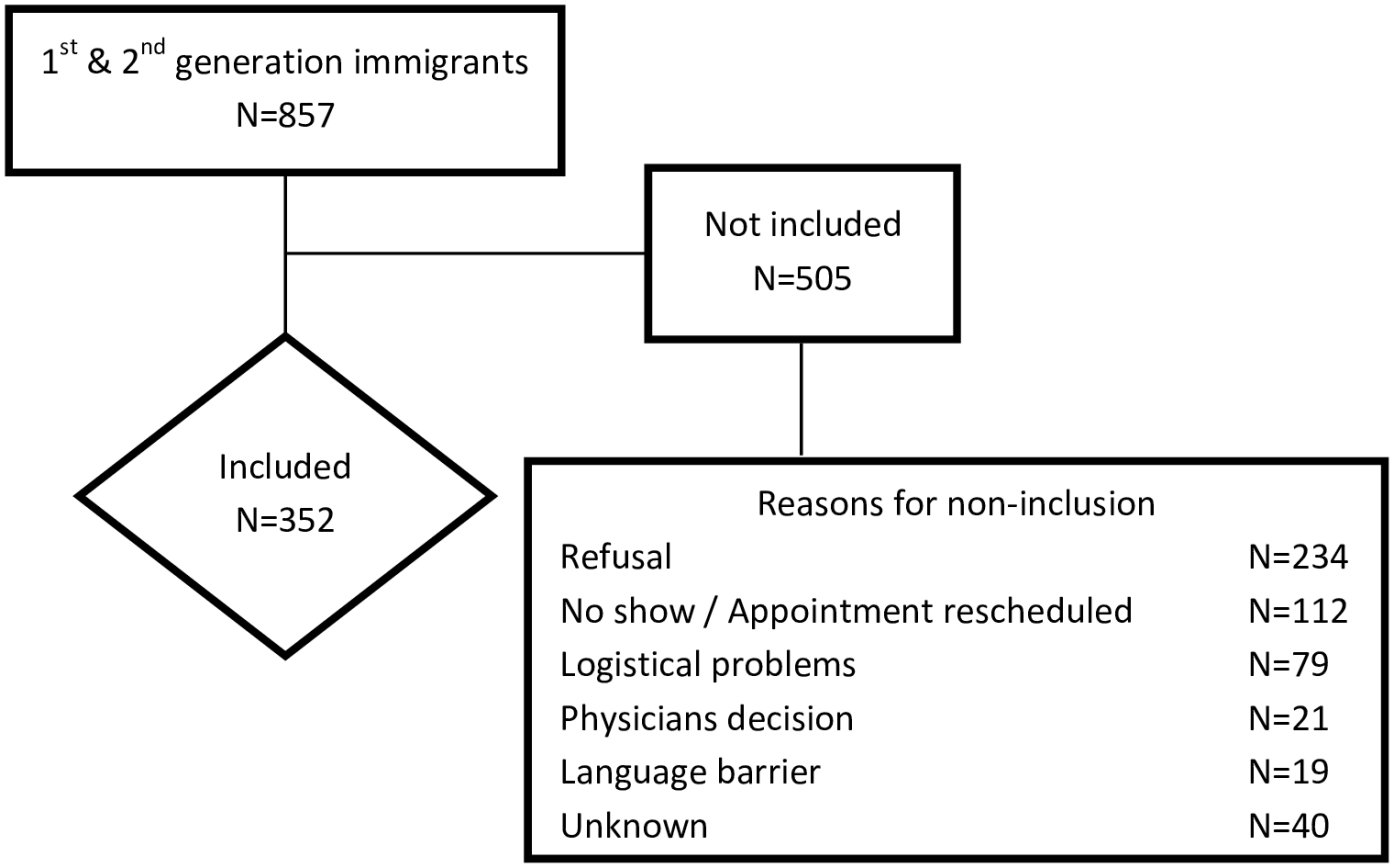
Flow diagram of patient inclusion. Legend. N = number of immigrant people living with HIV (PLWH).

Of the 857 immigrant PLWH attending the two Dutch treatment centers, 352 participated in this study (response = 41.1%). Reasons for non-inclusion were mainly refusal to participate due to, for example, fear of third party disclosure of HIV status, not wanting to talk about HIV, and lack of time (*n* = 234), and not having attended or having to reschedule the appointment (*n* = 112)(Fig 1). The only significant differences between the pool of eligible immigrant PLWH and the sample included were that the sample contained more cART experienced patients (86.2% vs. 77.4%) and less patients with a detectable HIV-RNA when cART experienced (11.0% vs. 17.3%) (Table 2).

**Table 2:** Characteristics of eligible participants at baseline.

	All N = 857	Included N = 352	Not included N = 505	*P*
**Mean age, years (SD)**	41.4 (11.3)	41.8 (10.6)	41.1 (11.8)	0.37^c^
**Male sex (%)**	517 (60.3)	202 (57.4)	315 (62.4)	0.14^d^
**Receiving cART (%)**^a^				<0.01^d^
*> 6 months*	688 (81.0)	301 (86.2)	387 (77.4)	
*< 6 months*	86 (10.1)	22 (6.3)	64 (12.8)	
*No cART*	75 (8.8)	26 (7.4)	49 (9.8)	
**CD4 cell count (%)**				0.15^d^
*<200 cells/mm*^*3*^	55 (6.4)	19 (5.4)	36 (7.1)	
*200–349 cells/mm*^*3*^	121 (14.1)	42 (11.9)	79 (15.6)	
*>350 cells/mm*^*3*^	681 (79.5)	291 (82.7)	390 (77.2)	
**HIV-2 (%)**	17 (2.0)	5 (1.4)	12 (2.4)	0.32^d^
**Plasma HIV-RNA > 50 copies/ml (%)**^b^	100 (14.5)	33 (11.0)	67 (17.3)	0.02^d^
**Region of origin (%)**				0.47^d^
*Sub Saharan Africa*	330 (38.5)	142 (40.3)	188 (37.2)	
*Caribbean*	180 (21.0)	67 (19.0)	113 (22.4)	
*Latin America*	164 (19.1)	72 (20.5)	92 (18.2)	
*Other*	183 (21.4)	71 (20.2)	112 (22.2)	

SD, standard deviation; cART, combination Antiretroviral Therapy.

^a^ Eight patients were previously treated with cART due to an acute HIV-infection and excluded from this analyses (N = 849).

^b^ When cART experienced (N = 688).

^c^ T-test.

^d^ Chi-square.

Table 3 presents participant characteristics of the included sample of immigrant PLWH. More than half (57.7%) were men. Most were first generation immigrants (94.6%) and most originated from Sub Saharan Africa (40.6%), followed by Latin America (22.7%), the Caribbean (19.6%), and then regions categorized as ‘other’ (17.0%). About two-thirds (62.5%) self-identified as heterosexual. In terms of their living situation, 39.2% reported living with family, 36.9% lived alone, and 16.2% were single parents. The majority had children (57.7%). Educational attainment varied from having attended no formal education or only primary school (23.3%*)*, to having attended secondary school (31.0%), higher vocational school (24.4%), or university (20.7%). In terms of employment, 43.5% were in paid employment, 25.9% were unemployed, 10.5% were on sick leave, and 20.2% reported having some other form of employment status.

**Table 3:** Socio-demographic characteristics and psychosocial variables.

	All N = 352	Subjects on cART^a^^,^^b^		*P*
		Adherent N = 139	Non-adherent N = 159	
**HIV-RNA >50 copies/ml (%)**	33 (11.0)^a^	9 (6.5)^c^	23 (14.5)^c^	<0.05^d^
**Age <35 years (%)**	95 (27.0)	25 (18.0)	34 (21.4)	0.46^d^
**Male gender (%)**	202 (57.4)	80 (57.6)	90 (56.6)	0.87^d^
**1**^**st**^ **generation immigrant (%)**	333 (94.6)	133 (95.7)	153 (96.2)	0.81^d^
**Region of origin (%)**				0.14^d^
*Sub Saharan Africa*	143 (40.6)	52 (37.4)	72 (45.3)	
*Caribbean*	69 (19.6)	26 (18.7)	36 (22.6)	
*Latin America*	80 (22.7)	30 (21.6)	30 (18.9)	
*Other*	60 (17.0)	31 (22.3)	21 (13.2)	
**Sexual orientation (%)**				0.55^e^
*Heterosexual*	220 (62.5)	87 (62.6)	107 (67.3)	
*Homosexual/Bisexual*	121 (34.4)	48 (34.5)	45 (28.3)	
*Does not know*	8 (2.3)	4 (2.9)	4 (2.5)	
**Living situation (%)**				0.49^d^
*With family*	138 (39.2)	61 (43.9)	57 (35.8)	
*Alone*	130 (36.9)	49 (35.3)	60 (37.7)	
*Single parent*	57 (16.2)	21 (15.1)	29 (18.2)	
*Other*	27 (7.7)	8 (5.8)	13 (8.2)	
**Children (%)**	203 (57.7)	83 (59.7)	102 (64.2)	0.48^d^
**Educational attainment (%)**				<0.05^d^
*No formal education / Primary school*	82 (23.3)	25 (18.0)	52 (32.7)	
*Secondary school*	109 (31.0)	45 (32.4)	48 (30.2)	
*Higher vocational school*	86 (24.4)	37 (26.6)	31 (19.5)	
*University*	73 (20.7)	32 (23.0)	26 (16.4)	
**Employment status (%)**				<0.05^d^
*Paid employment*	153 (43.5)	68 (48.9)	52 (32.7)	
*Unemployed*	91 (25.9)	27 (19.4)	56 (35.2)	
*On sick leave*	37 (10.5)	16 (11.5)	16 (10.1)	
*Other*	71 (20.2)	28 (20.1)	35 (22.0)	
**Alcohol (%)**				
*Alcohol in the past 30 days*	198 (56.3)	81 (58.3)	83 (52.2)	0.29^d^
*Alcohol use ≥3 days/week*	58 (16.5)	17 (12.2)	30 (18.9)	0.12^d^
**Drugs (%)**				
*Drugs in the past 30 days*	61 (17.3)	20 (14.4)	28 (17.6)	0.45^d^
*Drugs use ≥3 days/week*	36 (10.2)	10 (7.2)	19 (11.9)	0.17^d^
**Psychosocial variables (median, IQR)**^g^				
*Social support*	75 (43.8–90.6)	81.3 (50.0–96.9)	65.6 (37.5–85.9)	<0.001^f^
*Int*. *HIV-related stigma*	15 (12–19)	13 (12–17)	16 (12–19)	0.001^f^
*Adherence self-efficacy*	105 (89.8–116)	112.5 (98.8–119)	100 (87–110.5)	<0.001^f^
*Quality of life (physical)*	52.3 (42.3–56.2)	53.7 (43.9–56.6)	50.2 (40.5–55.5)	<0.05^f^
*Quality of life (mental)*	48.5 (37.6–56.5)	52.6 (41.2–58.1)	46.3 (37.2–55.2)	<0.01^f^

IQR, Interquartile Range; cART, combination Antiretroviral Therapy; Int., Internalized.

^a^ Subjects >6 months on cART (N = 301).

^b^ Three participants had missing values for self-reported adherence and were therefore excluded from this analyses (N = 298).

^c^ For 1 subject self-reported adherence score was missing, (N = 32).

^d^ Chi-square.

^e^ Fisher-Freeman-Halton Exact.

^f^ Mann-Whitney Test.

^g^ Missing values: social support (N = 20), internalized HIV related stigma (N = 19), adherence self-efficacy (N = 66), physical quality of life (N = 8), mental quality of life (N = 8).

### Factors associated with self-reported non-adherence

Of the cART experienced participants, 53.4% were non-adherent (Table 3).

In terms of clinical characteristics, non-adherent participants were more likely to have a detectable HIV-RNA (14.5% vs. 6.5%, *P*<0.05).

In terms of demographic characteristics, non-adherent participants were more likely to have a lower educational attainment (32.7% vs. 18.0%, *P*<0.05) and less likely to have paid employment (32.7% vs. 48.9%, *P*<0.05).

In terms of psychosocial variables, non-adherent participants had lower median social support scores (65.6 vs. 81.3, *P*<0.001), higher median internalized HIV-related stigma scores (16 vs. 13, *P* = 0.001), lower median adherence self-efficacy scores (100 vs. 112.5, *P*<0.001), and lower median physical quality of life (50.2 vs. 53.7, *P*<0.05) and mental quality of life (46.3 vs. 52.6, *P*<0.01) scores.

In the univariable analyses reported in Table 4, having a detectable HIV-RNA (OR = 2.44; 95%CI: 1.09–5.48), not having attended formal education or only primary school (OR = 2.56; 95% CI: 1.27–5.18, vs. University), and being unemployed (OR = 2.71; 95% CI: 1.51–4.86, vs. having a paid job) were factors associated with non-adherence. Participants experiencing low social support (OR = 2.61; 95% CI: 1.61–4.23), high internalized HIV-related stigma (OR = 2.45; 95% CI: 1.52–3.95), low treatment adherence self-efficacy (OR = 3.24; 95% CI: 1.93–5.44), low physical quality of life (OR = 1.75; 95% CI: 1.09–2.78) or low mental quality of life (OR = 1.92; 95% CI: 1.21–3.07) were also more likely to be non-adherent to cART.

**Table 4:** Factors related to self-reported non-adherence in cART experienced patients^a^.

	Univariable Regression			Multivariable Regression		
Variable	OR	95% CI	*P*	OR	95% CI	*P*
**HIV-RNA**						
*<50 copies/ml*	1			1		
*>50 copies/ml*	2.44	1.09–5.48	<0.05	2.53	0.91–7.06	0.08
**Age**						
*≥35 years*	1					
*<35 years*	1.24	0.69–2.21	0.46			
**Gender**						
*Male*	1					
*Female*	1.04	0.66–1.65	0.87			
**1**^**st**^ **generation immigrant**						
*No*	1					
*Yes*	1.15	0.36–3.65	0.81			
**Region of origin**						
*Sub Saharan Africa*	1			1		
*Caribbean*	1.00	0.54–1.86	1.00	1.72	0.75–3.93	0.19
*Latin America*	0.72	0.39–1.34	0.30	1.36	0.58–3.20	0.49
*Other*	0.49	0.25–0.95	<0.05	0.94	0.36–2.44	0.89
**Sexual orientation**						
*Homosexual /Bisexual*	1					
*Heterosexual*	1.31	0.79–2.15	0.28			
*Does not know*	1.07	0.25–4.52	0.93			
**Living situation**						
*With family*	1					
*Single parent*	1.48	0.76–2.88	0.25			
*Alone*	1.31	0.78–2.21	0.31			
*Other*	1.74	0.67–4.51	0.26			
**Educational attainment**						
*University*	1			1		
*Higher vocational school*	1.03	0.51–2.09	0.93	1.03	0.42–2.52	0.95
*Secondary school*	1.31	0.68–2.54	0.42	0.71	0.29–1.71	0.45
*No formal educ*.*/ Prim*. *school*	2.56	1.27–5.18	<0.01	3.25	1.28–8.26	<0.05
**Employment status**						
*Paid employment*	1			1		
*Unemployed*	2.71	1.51–4.86	<0.01	1.98	0.87–4.49	0.10
*On sick leave*	1.31	0.59–2.86	0.50	0.51	0.16–1.58	0.24
*Other*	1.64	0.88–3.02	0.12	1.19	0.50–2.84	0.69
**Alcoholic beverage <30 days**						
*No*	1					
*Yes*	0.78	0.49–1.24	0.29			
**Alcohol use ≥3 days per week**						
*No*	1					
*Yes*	1.67	0.88–3.18	0.12	1.66	0.70–3.92	0.25
**Drugs use < 30 days**						
*No*	1					
*Yes*	1.27	0.68–2.38	0.45			
**Social support**						
*High social support*	1			1		
*Low social support*	2.61	1.61–4.23	<0.001	2.56	1.37–4.82	<0.01
**Internalized HIV-related stigma**						
*Low internalized stigma*	1			1		
*High internalized stigma*	2.45	1.52–3.95	<0.001	1.82	0.97–3.43	0.06
**Self-efficacy**						
*High self-efficacy*	1			1		
*Low self-efficacy*	3.24	1.93–5.44	<0.001	2.99	1.59–5.64	<0.01
**Quality of life**						
*High physical QoL*	1			1		
*Low physical QoL*	1.75	1.09–2.78	<0.05	1.49	0.78–2.88	0.23
*High mental QoL*	1			1		
*Low mental QoL*	1.92	1.21–3.07	<0.01	1.24	0.65–2.38	0.52

Educ, Education; Prim, Primary; QoL, Quality of Life.

^a^All variables with a *P*<0.15 in the univariable analyses were submitted in multivariable analyses.

Several variables predicting non-adherence persisted in the multivariable analyses (Table 4). These were: not having attended formal education or only primary school (OR = 3.25; 95% CI: 1.28–8.26, vs. University), experiencing low social support (OR = 2.56; 95% CI: 1.37–4.82), and having low treatment adherence self-efficacy (OR = 2.99; 95% CI: 1.59–5.64). Having a detectable HIV-RNA and experiencing high internalized HIV-related stigma were also marginally associated with non-adherence in the multivariable analyses (OR = 2.53; 95% CI: 0.91–7.06 and OR = 1.82; 95% CI: 0.97–3.43, respectively). When participants classified as ‘adherent’ based on Q1, Q2 and Q3 responded with ‘I’m not sure’ to Q4 were classified as ‘non-adherent’ (N = 2), the association between a detectable HIV-RNA and non-adherence was statistically significant (OR = 3.17; 95%CI 1.10–9.11). Additional analyses showed an association between non-adherence and having a HIV-RNA >400 copies/ml (OR = 5.59; 95%CI 1.23–25.44) (S5 Table).

## Discussion

In this study, we assessed risk factors for non-adherence to cART among immigrant PLWH in the Netherlands. The results demonstrated that non-adherence to cART is associated with a lower educational attainment, experiencing low social support, and having low HIV treatment adherence self-efficacy. A detectable HIV-RNA and experiencing high internalized HIV-related stigma were also marginally associated with adherence in the multivariable analyses.

These findings are in line with those of other studies. For example, in a recent meta-analysis conducted by Langebeek and colleagues, social support, HIV-related stigma, and adherence self-efficacy were reported to be strongly associated with adherence to cART [12]. In addition, O’Connor and colleagues previously demonstrated an association between higher educational attainment and better adherence [27] while Glass and colleagues found that having only a basic education versus having higher education is a risk factor for worsening adherence over time [28]. Perhaps PLWH with greater educational attainment have better knowledge of HIV and thus realize the importance of being adherent to cART.

Self-reports are widely used in other studies of adherence and self-reported adherence has been found to be associated with HIV-RNA in multiple studies [29]. In our study, we combined four questions to determine self-reported adherence and conducted analyses using various cut-off points such that the criteria for adherence were either stricter or less strict (S2 Table). These analyses yielded the same predictors for non-adherence as those presented in this paper. We are, therefore, confident that the self-reported measure of adherence employed in our study was an appropriate measure for determining risk factors for adherence.

Not completely in line with previous studies demonstrating that HIV-related stigma is a predictor of adherence [6, 12], in our study, internalized HIV-related stigma was only marginally significant in the prediction of adherence. Our study, in its reliance on voluntary participation, may have recruited a sample with less internalized stigma than the eligible study population (stigma may very well have been a reason for non-participation and for non-adherence) thus yielding this only marginally significant association.

In addition to exploring risk factors for non-adherence, our study documented adherence and the extent to which immigrant PLWH have a detectable HIV-RNA. In accordance with previous research [4–6], we found that, compared to the general HIV population in the Netherlands whereby 91% of all PLWH on cART were virologically suppressed in 2014 [1], the eligible study population of immigrant PLWH contained more PLWH with detectable HIV-RNA. In fact, HIV-RNA was detectable in 14.5%. This is relatively in line with a previous study conducted in the Netherlands showing that 15% of immigrant PLWH had a detectable HIV-RNA [6]. However, the percentage of PLWH with detectable HIV-RNA was lower in our study sample (11.0%). A possible explanation for this discrepancy is that those who voluntarily participated represent a somewhat better performing patient population. If this is the case, our findings regarding risk factors for non-adherence may be even more relevant in the broader immigrant PLWH population. Another possible explanation is that current cART regimes are more effective than previous regimes, even when adherence is not optimal [30]. This contention appears to, at least in part, be supported by the fact that, in our study, 51.1% of the participants with an undetectable HIV-RNA were non-adherent compared to 71.9% of the participants with a detectable HIV-RNA. This, however, does not, in any way, justify non-adherence as non-adherence predisposes one to detectable HIV-RNA [6] and adherence levels to certain cART regimes of 70% still have been shown to promote residual HIV replication, even in the absence of virological rebound in plasma [31].

This study has a number of strengths and a few limitations that should be considered in the interpretation of the findings. One strength is that we successfully recruited and included 352 PLWH from a population that is generally very hard to reach and, in doing so, collected substantial data on their clinical, socio-demographic, and psychosocial characteristics. This is, to our knowledge, the largest sample of immigrant PLWH recruited for a study on risk factors for non-adherence to cART in the Netherlands to date. A second strength is that, in measuring adherence, we included both objective (biomarkers) and subjective (self-reported items) measures.

A possible limitation is that our sample may not fully represent the broader immigrant PLWH population in the Netherlands. Our response rate was 41.1% and our sample contained a lower percentage of cART experienced patients with a detectable HIV-RNA than in the eligible study population indicating the possibility of selection bias in the main analysis. As such, our results may, in fact, underestimate the predictive value of the risk factors for non-adherence found. Given reasons for non-participation reported, it is quite possible that those refusing to participate experience more HIV-related stigma and related disclosure concerns than those who did participate thereby leading to an underestimation of the predictive value of HIV-related stigma. This is supported by findings from other studies indicating significant perceived stigma and disclosure concerns due to anticipated stigma among immigrant PLWH in the Netherlands [15, 32]. Another limitation is that data from 18.8% of the sample were missing for treatment adherence self-efficacy. Participants struggled to understand the items in this scale, despite assistance. A final limitation is the cross-sectional nature of this study. Because treatment with cART is currently a lifelong commitment and adherence is complex, it is possible that predictors of adherence change over time. We, therefore, suggest that future studies longitudinally measure adherence and its associated risk factors on multiple occasions over a longer period of time.

## Conclusion

In conclusion, our findings provide important insights for the development of theory and evidence-based interventions that aim to improve adherence to cART in immigrant PLWH. In addition to monitoring patients with low educational attainment, low social support, and low treatment adherence self-efficacy, we recommend the development of interventions that aim to increase social support and treatment adherence self-efficacy while considering the impact of education on adherence. This could potentially take the form of more intensive counselling and formal support from HIV care providers or informal support by trained peers. Also, given the possibility that our findings underestimated the effects of HIV-related stigma on adherence, interventionists may want to consider engaging in efforts to reduce or combat the effects of HIV-related stigma. Interventions seeking to improve adherence should subsequently be evaluated for effect, while controlling for variability in the standard of care provided to control groups as this can influence the effect sizes of behavior change interventions [33, 34]. Also, in the development of such interventions, it is imperative that cultural background of immigrant PLWH and possible challenges such as language barriers and cultural appropriateness be considered.

## Supporting Information

S1 Table Self-reported adherence questions. (PDF)

S2 Table Adherence measures. (PDF)

S3 Table Adherence I. Factors related to self-reported adherence in cART experienced patients. Educ, Education; Prim, Primary; QoL, Quality of Life. ^a^All variables with a *P*<0.15 in the univariable analyses were submitted in multivariable analyses.
(PDF)

S4 Table Adherence III. Factors related to self-reported adherence in cART experienced patients. Educ, Education; Prim, Primary; QoL, Quality of Life. ^a^All variables with a *P*<0.15 in the univariable analyses were submitted in multivariable analyses.
(PDF)

S5 Table Association between adherence and HIV-RNA. (PDF)
