# Supplementary material for: Risk Factors for Non-Adherence to cART in Immigrants with HIV Living in the Netherlands: Results from the ROtterdam ADherence (ROAD) Project
Source: PLoS One. 2016 Oct 5;11(10):e0162800. doi: 10.1371/journal.pone.0162800 (PMC5051866; doi:10.1371/journal.pone.0162800)
Supplement: S1 Table — (PDF) [file pone.0162800.s001.pdf]

Self-reported adherence questions

**Questions**	**Response options**
1. Thinking about the past 4 weeks, how would you rate your ability to take all your medications as your doctor prescribed them?	A. Very poor<br>B. Poor<br>C. Fair<br>D. Good<br>E. Very good<br>F. Excellent
2. Thinking about the past 4 weeks, how often did you take all your HIV antiretroviral medications as your doctor prescribed them?	A. None of the time<br>B. A little of the time<br>C. A good bit of the time<br>D. Most of the time<br>E. All of the time
3. How many days in the past week did you take all anti-HIV medicines that were prescribed?	A. Not one day<br>B. 1 or 2 days<br>C. 3 or 4 days<br>D. 5 or 6 days<br>E. All 7 days
4. When was the last time you missed any of you anti-HIV medications?	A. Within the past week<br>B. 1-2 weeks ago<br>C. 2-4 weeks ago<br>D. 1-3 months ago<br>E. More than 3 months ago<br>F. Never missed
