# Supplementary material for: Risk Factors for Non-Adherence to cART in Immigrants with HIV Living in the Netherlands: Results from the ROtterdam ADherence (ROAD) Project
Source: PLoS One. 2016 Oct 5;11(10):e0162800. doi: 10.1371/journal.pone.0162800 (PMC5051866; doi:10.1371/journal.pone.0162800)
Supplement: S2 Table — (PDF) [file pone.0162800.s002.pdf]

Adherence measures

**Measures**	Q1	Q2	Q3	Q4	Result
**Adherence I**	A, B, C, D, E	A, B, C, D	A, B, C, D	A, B, C	Non-adherent
	F	E	E	D, E, F	Adherent
**Adherence II**	A, B, C, D	A, B, C, D	A, B, C, D	A, B, C	Non-adherent
	E, F	E	E	D, E, F	Adherent
**Adherence III**	A, B, C, D	A, B, C, D	A, B, C, D	A, B	Non-adherent
	E, F	E	E	C, D, E, F	Adherent
