# Supplementary material for: Risk Factors for Non-Adherence to cART in Immigrants with HIV Living in the Netherlands: Results from the ROtterdam ADherence (ROAD) Project
Source: PLoS One. 2016 Oct 5;11(10):e0162800. doi: 10.1371/journal.pone.0162800 (PMC5051866; doi:10.1371/journal.pone.0162800)
Supplement: S3 Table — Educ, Education; Prim, Primary; QoL, Quality of Life. aAll variables with a P<0.15 in the univariable analyses were submitted in multivariable analyses. (PDF) [file pone.0162800.s003.pdf]

Adherence I. Factors related to self-reported non-adherence in cART experienced patients[a]

	Univariable Regression			Multivariable Regression		
**Variable**	**OR**	**95% CI**	***P***	**OR**	**95% CI**	***P***
**HIV-RNA**						
<50 copies/ml	1			1		
>50 copies/ml	2.05	0.86-4.92	0.11	2.03	0.67-6.14	0.21
**Age**						
≥35 years	1			1		
<35 years	1.74	0.92-3.31	<0.1	2.13	0.92-4.96	0.08
**Gender**						
Male	1					
Female	1.11	0.68-1.79	0.68			
**1st generation immigrant**						
No	1					
Yes	0.61	0.16-2.31	0.47			
**Region of origin**						
Sub Saharan Africa	1			1		
Caribbean	0.83	0.43-1.59	0.57	1.21	0.50-2.94	0.67
Latin America	0.55	0.89-1.05	<0.1	0.93	0.39-2.26	0.88
Other	0.46	0.24-0.89	<0.05	0.99	0.37-2.66	0.98
**Sexual orientation**						
Homosexual / bisexual	1					
Heterosexual	1.34	0.80-2.24	0.26			
Does not know	1.10	0.25-4.89	0.89			
**Living situation**						
With family	1			1		
Single parent	2.33	1.11-4.90	<0.05	2.27	0.80-6.44	0.12

Alone	1.49	0.87-2.56	0.15	0.99	0.46-2.15	0.99
Other	1.84	0.67-5.07	0.24	1.66	0.41-6.77	0.48
**Educational attainment**						
University	1			1		
Higher vocational school	0.96	0.47-1.95	0.91	0.88	0.36-2.15	0.77
Secondary school	1.38	0.70-2.69	0.35	1.12	0.48-2.64	0.79
No formal educ. / Prim. school	2.89	1.36-6.16	<0.01	5.41	1.84-15.93	<0.01
**Employment status**						
Paid employment	1			1		
Unemployed	2.25	1.21-4.18	<0.05	1.32	0.55-3.16	0.53
On sick leave	1.19	0.53-2.66	0.66	0.41	0.12-1.41	0.16
Other	1.33	0.71-2.51	0.38	0.92	0.37-2.31	0.87
**Alcoholic beverage <30 days**						
No	1					
Yes	0.90	0.56-1.45	0.67			
**Alcohol use ≥3 days per week**						
No	1			1		
Yes	2.57	1.19-5.55	<0.05	2.57	0.98-6.77	0.06
**Drugs use < 30 days**						
No	1					
Yes	1.09	0.57-2.09	0.80			
**Social support (75)**						
High social support	1			1		
Low social support	2.38	1.44-3.96	<0.01	2.41	1.19-4.89	<0.05
**Internalized HIV-related stigma**						
Low internalized stigma	1			1		
High internalized stigma	1.97	1.19-3.25	<0.01	1.35	0.69-2.63	0.38

**Self-efficacy (105)**						
High self-efficacy	1			1		
Low self-efficacy	3.96	2.25-6.95	<0.001	3.24	1.67-6.31	<0.001
**Quality of life**						
High physical QoL	1			1		
Low physical QoL	1.66	1.02-2.69	<0.05	1.64	0.81-3.30	0.17
High mental QoL	1			1		
Low mental QoL	1.79	1.09-2.93	<0.05	0.97	0.49-1.92	0.93

Educ, Education; Prim, Primary; QoL, Quality of Life.

[a]All variables with a *P*<0.15 in the univariable analyses were submitted in multivariable analyses.
