# Supplementary material for: Risk Factors for Non-Adherence to cART in Immigrants with HIV Living in the Netherlands: Results from the ROtterdam ADherence (ROAD) Project
Source: PLoS One. 2016 Oct 5;11(10):e0162800. doi: 10.1371/journal.pone.0162800 (PMC5051866; doi:10.1371/journal.pone.0162800)
Supplement: S4 Table — Educ, Education; Prim, Primary; QoL, Quality of Life. aAll variables with a P<0.15 in the univariable analyses were submitted in multivariable analyses. (PDF) [file pone.0162800.s004.pdf]

Adherence III. Factors related to self-reported non-adherence in cART experienced patients[a]

	Univariable Regression			Multivariable Regression		
**Variable**	**OR**	**95% CI**	***P***	**OR**	**95% CI**	***P***
**HIV-RNA**						
<50 copies/ml	1			1		
>50 copies/ml	2.41	1.09-5.28	<0.05	2.44	0.90-6.57	0.08
**Age**						
≥35 years	1			1		
<35 years	1.35	0.76-2.39	0.31			
**Gender**						
Male	1					
Female	1.12	0.71-1.77	0.64			
**1st generation immigrant**						
No	1					
Yes	1.42	0.44-4.58	0.56			
**Region of origin**						
Sub Saharan Africa	1			1		
Caribbean	0.97	0.52-1.79	0.92	1.93	0.82-4.50	0.13
Latin America	0.65	0.35-1.21	0.18	1.34	0.53-3.42	0.54
Other	0.46	0.36-0.89	<0.05	1.04	0.38-2.86	0.94
**Sexual orientation**						
Homosexual / bisexual	1			1		
Heterosexual	1.57	0.95-2.58	<0.1	1.25	0.58-2.70	0.57
Does not know	1.39	0.33-5.88	0.66	2.21	0.27-17.8	0.46
**Living situation**						
With family	1					
Single parent	1.33	0.69-2.58	0.40			

Alone	1.34	0.79-2.27	0.27			
Other	1.99	0.77-5.17	0.16			
**Educational attainment**						
University	1			1		
Higher vocational school	0.99	0.49-2.02	0.98	0.89	0.35-2.23	0.79
Secondary school	1.33	0.69-2.58	0.40	0.71	0.29-1.73	0.46
No formal educ. / Prim. school	2.62	1.30-5.29	<0.01	2.83	1.12-7.14	<0.05
**Employment status**						
Paid employment	1			1		
Unemployed	2.89	1.62-5.17	<0.001	2.05	0.91-4.63	0.08
On sick leave	1.37	0.63-3.01	0.43	0.53	0.17-1.64	0.26
Other	1.71	0.92-3.16	<0.1	0.99	0.41-2.38	0.97
**Alcoholic beverage <30 days**						
No	1					
Yes	0.81	0.51-1.27	0.35			
**Alcohol use ≥3 days per week**						
No	1					
Yes	1.43	0.76-2.68	0.27			
**Drugs use < 30 days**						
No	1					
Yes	1.35	0.72-2.51	0.35			
**Social support**						
High social support	1			1		
Low social support	2.73	1.68-4.42	<0.001	2.76	1.47-5.19	<0.01
**Internalized HIV-related stigma**						
Low internalized stigma	1			1		
High internalized stigma	2.12	1.32-3.41	<0.01	1.56	0.83-2.92	0.17

**Self-efficacy**						
High self-efficacy	1			1		
Low self-efficacy	3.07	1.84-5.15	<0.001	2.99	1.57-5.73	<0.01
**Quality of life**						
High physical QoL	1			1		
Low physical QoL	1.64	1.03-2.61	<0.05	1.30	0.68-2.50	0.43
High mental QoL	1			1		
Low mental QoL	1.89	1.19-3.01	<0.01	1.18	0.62-2.53	0.62

Educ, Education; Prim, Primary; QoL, Quality of Life.

[a]All variables with a *P*<0.15 in the univariable analyses were submitted in multivariable analyses.
