# Supplementary material for: Risk Factors for Non-Adherence to cART in Immigrants with HIV Living in the Netherlands: Results from the ROtterdam ADherence (ROAD) Project
Source: PLoS One. 2016 Oct 5;11(10):e0162800. doi: 10.1371/journal.pone.0162800 (PMC5051866; doi:10.1371/journal.pone.0162800)
Supplement: S5 Table — (PDF) [file pone.0162800.s005.pdf]

Association between adherence and HIV-RNA

**Variable**	**HIV-RNA**		OR	95% CI	P
**Adherence**	*<400 copies/ml*	*≥400 copies/ml*			
Adherent	137	2	1		
Non-adherent	147	12	5.59	1.23-25.44	0.26
